# 4-Octyl itaconate reduces influenza A replication by targeting the nuclear export protein CRM1

**DOI:** 10.1128/jvi.01325-23

**Published:** 2023-10-12

**Authors:** Pau Ribó-Molina, Hauke J. Weiss, Balasubramanian Susma, Stefan van Nieuwkoop, Leentje Persoons, Yunan Zheng, Melanie Ruzek, Dirk Daelemans, Ron A. M. Fouchier, Luke A. J. O'Neill, Bernadette G. van den Hoogen

**Affiliations:** 1 Department of Viroscience, Erasmus Medical Center, Rotterdam, the Netherlands; 2 School of Biochemistry and Immunology, Trinity Biomedical Sciences Institute, Trinity College Dublin, Dublin, Ireland; 3 Laboratory of Virology and Chemotherapy, KU Leuven Department of Microbiology, Immunology and Transplantation, Rega Institute, KU Leuven, Leuven, Belgium; 4 AbbVie Bioresearch Center, Worcester, Massachusetts, USA; St. Jude Children's Research Hospital, Memphis, Tennessee, USA

**Keywords:** influenza virus, CRM1, itaconate, 4-OI, antiviral

## Abstract

In recent years, especially since the outbreak of the severe acute respiratory syndrome coronavirus 2 pandemic, the cell-permeable itaconate derivative 4-octyl itaconate (4-OI) has gained traction as a potential antiviral agent. Here, we demonstrate that 4-OI inhibits replication of multiple influenza A viruses (IAV) by restricting nuclear export of viral ribonucleoproteins, a key step in the IAV replication cycle. This nuclear retention is achieved by deactivation and subsequent degradation of chromosomal maintenance 1 protein (CRM1), also known as exportin 1 (XPO1), a host cell protein exploited by IAV during replication. 4-OI-mediated deactivation of CRM1 resulted in the accumulation of the IAV nucleoprotein, the Rev protein of feline immunodeficiency virus, as well as the natural CRM1 cargos p53 and p65, in the nucleus of treated cells. Further mechanism of action studies revealed that, similar to known CRM1 inhibitors, 4-OI modifies a key cysteine in the cargo binding pocket of CRM1 at position 528 through an alkylation reaction called 2,3-dicarboxypropylation. Subsequent studies in a cell line in which the cysteine at position 528 in CRM1 protein was substituted by a serine confirmed that modification of this residue was indeed the cause for the observed inhibitory effect induced by 4-OI on CRM1 function. Overall, this study demonstrated a mechanism through which 4-OI directly interferes with the replication cycle of CRM1-dependent viruses, which contributes to the understanding of the antiviral and anti-inflammatory properties of this multifaceted immuno-metabolite.

**IMPORTANCE:**

Itaconate derivates, as well as the naturally produced metabolite, have been proposed as antivirals against influenza virus. Here, the mechanism behind the antiviral effects of exogenous 4-octyl itaconate (4-OI), a derivative of itaconate, against the influenza A virus replication is demonstrated. The data indicate that 4-OI targets the cysteine at position 528 of the CRM1 protein, resulting in inhibition of the nuclear export of viral ribonucleoprotein complexes in a similar manner as previously described for other selective inhibitors of nuclear export. These results postulate a mechanism not observed before for this immuno-metabolite derivative. This knowledge is helpful for the development of derivatives of 4-OI as potential antiviral and anti-inflammatory therapeutics.

## INTRODUCTION

Influenza virus is a negative-sense single-stranded RNA virus with a segmented genome, which belongs to the family of *Orthomyxoviridae*. Characteristically, human infections with influenza virus cause upper respiratory tract disease, with symptoms that vary from fever and muscle ache to cough or a sore throat, as well as fatigue (1). In humans, this disease is mostly caused by influenza A and B viruses (IAV and IBV, respectively), where severe symptoms can lead to lethal pneumonia during infections of the lower respiratory tract. Influenza viruses cause annual epidemics but can also lead to worldwide pandemics. When vaccines are not available, antivirals could be used to contain or limit their spread.

A metabolite that has recently been described as a potential antiviral is itaconate, which, alongside various derivatives, has proven to restrict replication of Zika virus (2), severe acute respiratory syndrome coronavirus 2 (SARS-CoV-2) (3), and IAV (4). Itaconate is generated from aconitate by the enzyme aconitate decarboxylase 1 (ACOD1), and expression of the Acod1 gene, also named immuno-responsive gene 1 (Irg1), is mostly restricted to cells of the myeloid lineage (5). Originally suggested as an anti-inflammatory metabolite, itaconate has multiple immunomodulatory properties, including impairment of glycolysis (6), inactivation of the NLRP3 inflammasome (7), activation of the activating transcription factor 3 (ATF3) (8), and activation of the nuclear factor erythroid2-related factor 2 (Nrf2) by introducing alkylation and thereby deactivation of the Kelch-like ECH-associated protein 1 (KEAP1) in a process called dicarboxypropylation (9). The latter has been suggested to be at least partly responsible for some of the antiviral properties of itaconate, as activation of this pathway has been associated with reduced viral replication of SARS-CoV-2 in treated cells (3). The mechanism through which Nrf2 activation restricts viral replication, however, remains unknown.

Being a negatively charged polar metabolite, itaconate has been proposed to be unable to spontaneously cross the cell membrane, prompting the design and use of cell-permeable itaconate derivatives such as dimethyl itaconate (DMI) (10) and 4-octyl-itaconate (4-OI) (9). These derivatives share the electrophilic properties of itaconate and were thought to be, at least in part, metabolized into itaconate. However, it appeared that DMI is quickly degraded and, in fact, not converted to itaconate (11), while 4-OI actually increased the levels of intracellular itaconate (9). Moreover, 4-OI seemed to have additional immunoregulatory effects that go beyond the properties of unmodified itaconate (12).

Recently, the antiviral properties of 4-OI against IAV were reported (4), but the mechanism of action remained elusive. Previously, other itaconate derivates have been suggested to restrict the nuclear export of viral ribonucleoproteins (vRNPs) (13). A shared property of itaconate and its derivatives is the capability to alkylate cysteine residues on certain proteins (6, 9, 14). Molecules such as leptomycin B (LMB) and the small-molecule inhibitors selinexor (KPT-330) and verdinexor have been described to alkylate the chromosomal maintenance 1 protein (CRM1) at cysteine 528, thereby inhibiting the export of proteins that contain a nuclear export signal (NES) (15 – 18). Here, we demonstrate that 4-OI alkylates CRM1 thereby affecting CRM1-mediated nuclear export of proteins with a NES, such as the IAV nucleoprotein (NP). These findings contribute to the understanding of the antiviral and anti-inflammatory properties of this multifaceted immuno-metabolite.

## RESULTS

### 4-Octyl itaconate impairs IAV replication in a dose-dependent manner

**Fig 1:**
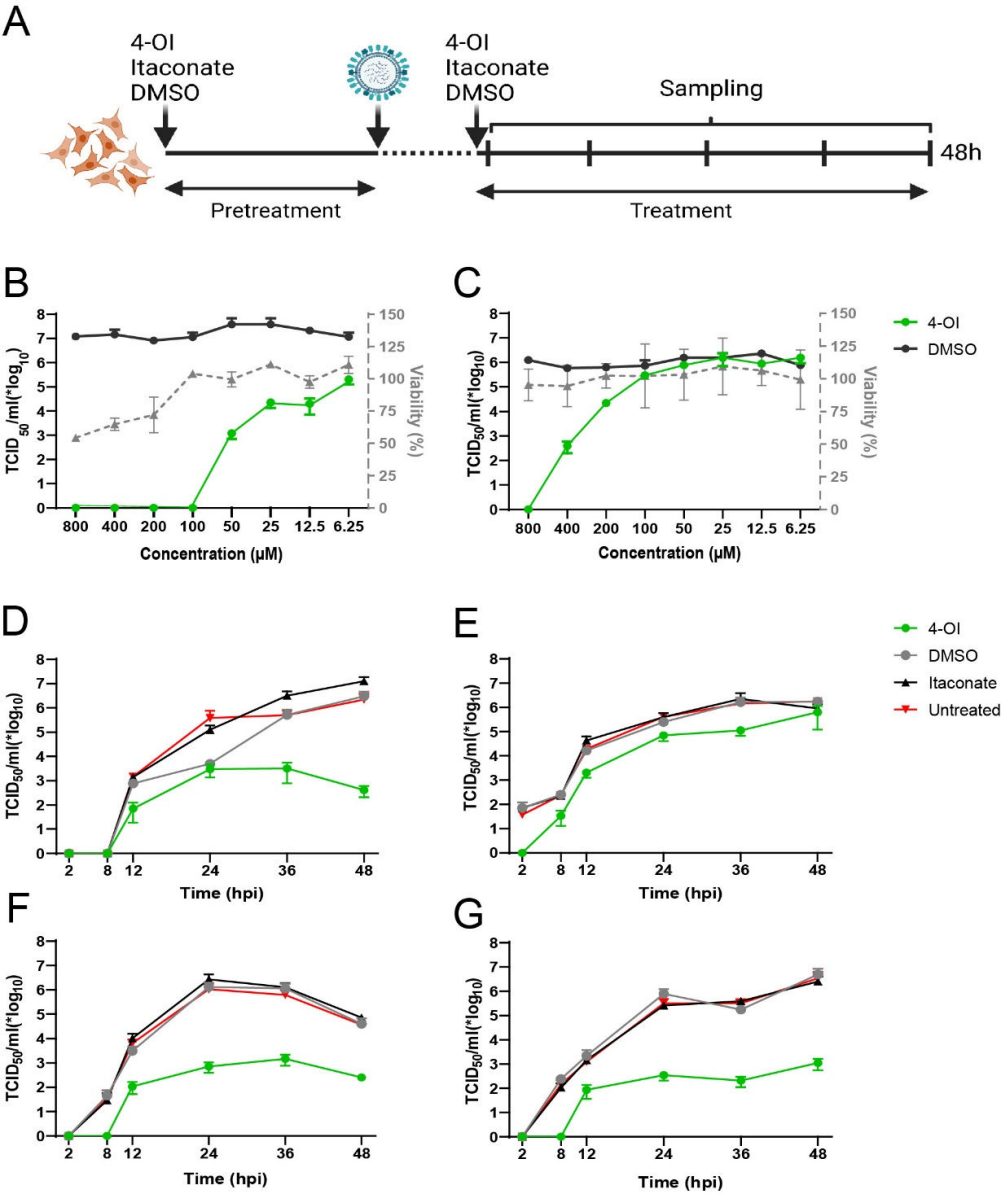
Effects of 4-OI on influenza A virus replication. (**A**) Outline of the treatment scheme. Epithelial cells were pretreated overnight with 4-OI, DMSO, or itaconate or were left untreated and were subsequently inoculated with IAV A/WSN/33. After 1 hour, inoculum was removed, and cells were washed. Medium with the corresponding treatments was added until sampling. Created with BioRender.com. (**B and C**) Dose-dependent effect of 4-OI compared to DMSO on virus titers (left Y axis) at 48 hours post inoculation in MDCK (B, multiplicity of infection [MOI] 0.01) and A549 (C, MOI 0.1) cells, with corresponding live/dead staining of 4-OI-treated cells normalized to DMSO measured using flow cytometry (right Y-axis). (**D and E**) Replication kinetics of IAV A/WSN/33 in MDCK (D, MOI 0.01) and A549 cells (E, MOI 0.1), treated with 4-OI, DMSO, or itaconate or left untreated. (**F and G**) Replication kinetics of IAV A/NL/602/09 H1N1 (F, MOI 0.01) and A/NL/213/03 H3N2 (G, MOI 0.01) in MDCK cells, treated with 4-OI, DMSO, or itaconate or left untreated. All kinetics are representative of three independent experiments and are depicted as mean ± SEM.

To investigate whether 4-OI had an impact on IAV replication, a treatment protocol was established with a short pre-treatment prior to virus inoculation, combined with treatment after inoculation (Fig. 1A). Maintenance of selection pressure with 4-OI treatment after inoculation was found essential to produce an effect on IAV replication (Fig. S1). A549 cells, as a model of human epithelial cells, and Madin-Darby canine kidney (MDCK) cells, as example of a highly permissive cell line to IAV, were used to optimize the dose range of the compound. At 48 hours after inoculation with influenza A/WSN/33 (IAV WSN), substantially lower virus titers were observed in treated MDCK (Fig. 1B) and A549 cells (Fig. 1C), compared to those in DMSO-treated cells, in a dose-dependent manner. While, in MDCK cells, antiviral effects were visible at 100 µM, higher concentrations were necessary to induce an antiviral effect in A549 cells. In parallel, cell viability was measured to determine the optimal concentration of 4-OI, normalized to DMSO. Treatment with 4-OI decreased the viability of MDCK cells at concentrations beyond 100 µM (Fig. 1B), whereas A549 cells seemed to be more tolerant (Fig. 1C).

Replication kinetics of IAV WSN were assessed in treated MDCK (Fig. 1D) and A549 cells (Fig. 1E), the maximum tolerated dose for MDCK cells of 100 µM. The effect of treatment with 100 µM 4-OI on the replication of IAV WSN was compared to that of treatment with the natural metabolite itaconate at 10 mM. Untreated and DMSO-treated cells were used as controls. Compared to replication in untreated cells, IAV WSN replication was decreased in both cell lines treated with 4-OI, while replication was not affected upon treatment with itaconate or DMSO. The antiviral effect of 4-OI against IAV was more profound in MDCK cells than in A549 cells, which correlates with the effectivity shown in Fig. 1C where higher doses of 4-OI than 100 µM were needed to achieve a profound antiviral effect against IAV.

To confirm the observations made for IAV WSN, the effect of 4-OI treatment on replication of two additional IAV strains was assessed in MDCK cells. Both IAV pandemic H1N1 (Fig. 1F) and H3N2 (Fig. 1G) replicated less efficiently in cells treated with 100 µM 4-OI, compared to untreated cells. In contrast, in cells treated with DMSO or itaconate, IAV replication was similar to that in untreated cells.

### Selective effect of 4-OI on IAV NP and CRM1 localization and expression levels

**Fig 2:**
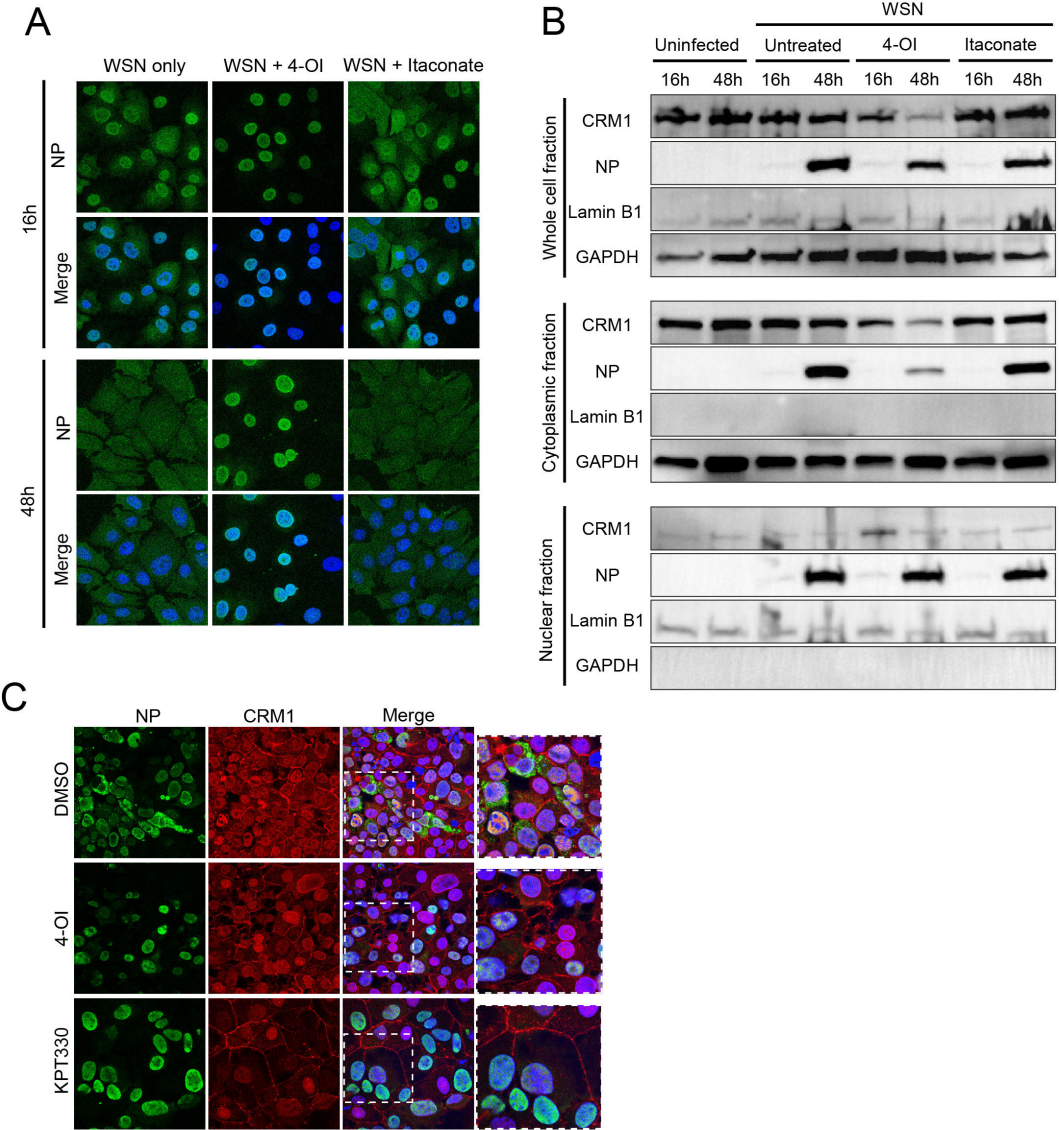
Effect of 4-OI on NP and CRM1 localization. (**A**) Confocal microscopy image of MDCK cells inoculated with IAV A/WSN/33 (MOI 0.01) treated with 4-OI, itaconate, or left untreated. At 16 and 48 hours post inoculation (hpi), cells were fixed and stained for NP (green), to assess cellular localization. Merged depicts NP (green) together with nuclear staining Hoechst (blue). Confocal images are representative of three individual experiments. (**B**) Protein expression levels of CRM1 and IAV NP in whole cell lysate, cytoplasmic, and nuclear fractions of uninfected MDCK cells and MDCK cells inoculated with IAV A/WSN/33 (MOI 0.01), without treatment or treated with 100 µM 4-OI and itaconate. Nuclear marker Lamin B1 and cytoplasmic marker GAPDH were included for comparison. Images are representative of three individual experiments. (**C**) Confocal microscopy image of MDCK cells inoculated with IAV A/WSN/33 (MOI 0.01) and treated with equimolar DMSO, 100 µM 4-OI, and 100 nM KPT-330. At 24 hpi, cells were fixed and stained for the NP protein (green), CRM1 (red), nuclear staining Hoechst (blue) shown in Merge. Confocal images are representative of three individual experiments.

Since replication of the IAV genome occurs in the nucleus, we assessed the effect of 4-OI on replication by studying the localization of NP of IAV WSN in 4-OI-treated MDCK cells. Untreated cells and cells treated with 10 mM itaconate were taken as control, and localization of NP was examined with confocal microscopy. Upon inoculation with IAV WSN (MOI of 0.01) of the 4-OI-treated cells, nuclear trapping of the NP protein was observed, which was not observed in untreated or itaconate-treated cells (Fig. 2A). To examine the effects at earlier time points after virus inoculation, cells were also inoculated at a higher MOI of 1, and cells were examined at 8 hours after inoculation. Subsequent confocal microscopy showed indeed nuclear trapping of the NP protein in treated cells. (Fig. S2A). These results suggest that 4-OI inhibits the transport of IAV NP from the nucleus into the cytoplasm of the infected cell.

CRM1-mediated export of IAV NP is known to be essential for IAV replication (17). Analysis of expression levels of CRM1 and IAV NP in 100 µM 4-OI-treated cells revealed lower CRM1 expression levels in whole-cell and cytoplasmic fractions in treated cells as compared to untreated or itaconate treated at 48 hours after inoculation with IAV WSN at an MOI of 0.01 (Fig. 2B). Interestingly, IAV NP expression levels in the cytoplasmic fraction of 4-OI-treated cells were lower than those in the cytoplasmic fraction of untreated cells or itaconate-treated infected cells, correlating with the trapping of IAV NP in the nucleus in 4-OI-treated cells as shown in Fig. 2A. A slight decrease of IAV NP was also observed in the whole-cell fraction of 4-OI-treated cells at 48 hours after inoculation with IAV WSN at an MOI of 0.01, compared to untreated or itaconate-treated cells. In contrast, IAV NP expression levels in the nuclear fraction were similar for 4-OI- and itaconate-treated and untreated IAV-infected cells. Examination at 8 hpi at the higher MOI of 1 revealed that cytoplasmic expression levels of IAV NP were below detection levels, while NP expression levels were slightly higher in the nuclear and whole-cell fraction of 4-OI-treated cells (Fig. S2B). At 8 hpi and at the higher MOI of 1, expression levels of CRM1 in the cytoplasm and the whole-cell fraction were slightly lower in 4-OI-treated cells compared to untreated cells (Fig. S2B).

To confirm the relationship between decreased CRM1 expression levels and decreased cytoplasmic IAV NP expression levels, the effect of KPT-330, a well-known inhibitor of CRM1-mediated export of NES containing proteins, on IAV NP localization was assessed. To this end, MDCK cells treated with 100 µM 4-OI were inoculated with IAV WSN at an MOI of 0.01 and compared with 100 nM KPT-330-treated and KPT-330-inoculated cells. Both in 4-OI- and KPT-330-treated and IAV-inoculated cells, nuclear trapping of the IAV NP was observed, in contrast to DMSO-treated infected cells. CRM1 expression levels were higher in the nucleus of 4-OI- and KPT-330-treated cells than in those of DMSO-treated cells at 24 hpi (Fig. 2C). The effect of 4-OI on NP nuclear localization and CRM1 decrease was also observed at 8 hpi after inoculation at the higher MOI of 1; however, expression levels of NP were lower than at later timepoints post inoculation (Fig. S2C). Overall, the nuclear trapping of the IAV NP protein upon 4-OI treatment was similar as observed upon treatment with KPT-330, confirming an effect on CRM1-mediated protein trafficking from the nucleus to the cytoplasm.

### 4-OI inhibits CRM1 activity as a nuclear cargo exporter

**Fig 3:**
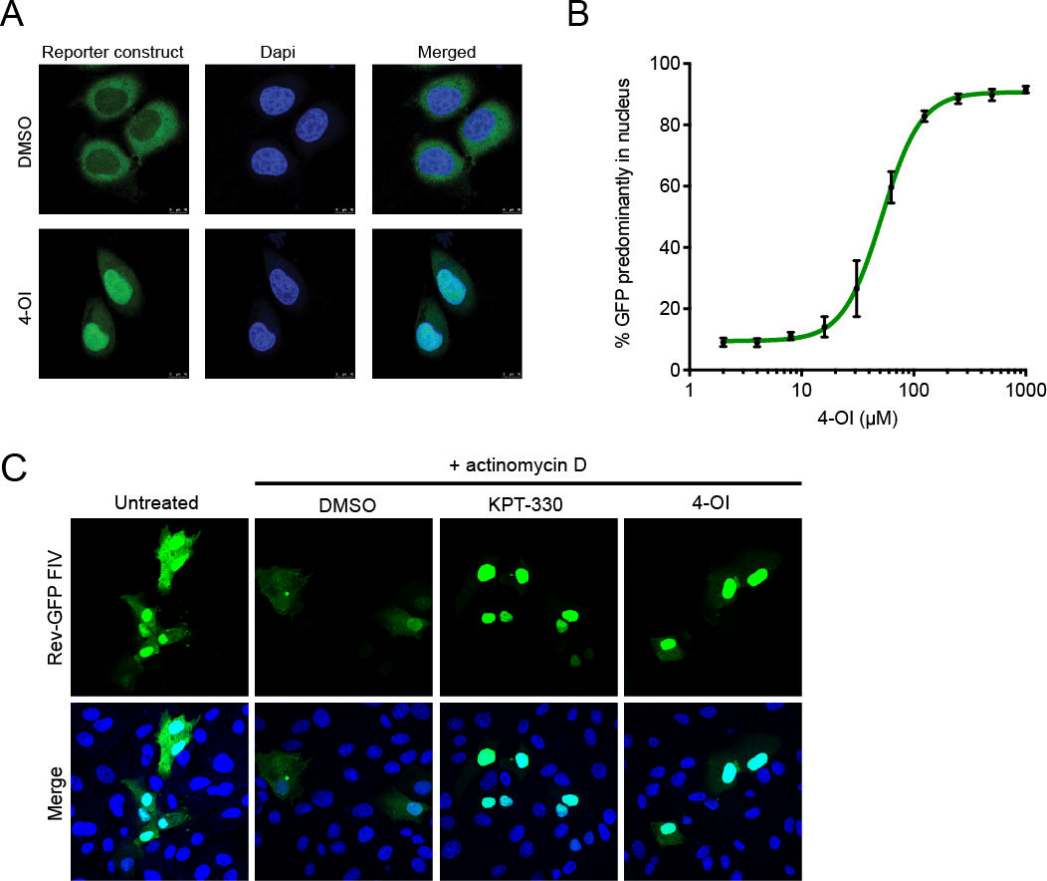
Impact of 4-OI on CRM1-dependent proteins. (**A**) The effect of treatment with 4-OI in a reporter HeLa cell line stably expressing a CRM1-dependent GFP reporter protein. Cells were treated with 100 µM 4-OI or DMSO for 24 hours, and the subcellular location was assessed by confocal microscopy. Confocal images are representative of three independent experiments. (**B**) Quantification of results from panel A. Cells were treated with indicated concentrations of 4-OI for 24 hours, and the percentage of cells with a predominantly nuclear localization of the GFP reporter protein was assessed. The experiment was conducted three times, and results are depicted as mean ± SD. (**C**) Confocal microscopy image of Vero-118 cells transfected with FIV Rev-GFP (green). Cells were left untreated or treated with actinomycin D simultaneously to DMSO, 100 µM 4-OI, or 100 nM KPT-330. Merge images include nuclear staining with Hoechst (blue). Confocal images are representative of three individual experiments.

To further investigate the effect of 4-OI on CRM1 activity, a CRM1-dependent GFP cargo assay was employed to visualize and quantify the effectivity of 4-OI as an inhibitor of CRM1 activity. To this end, HeLa cells stably expressing a green fluorescent protein (GFP) containing the CRM1-dependent NES from protein kinase inhibitor (PKI) were used. This GFP reporter protein shuttles between the nucleus and the cytoplasm and accumulates in the nucleus upon inactivation of CRM1 (19). The HeLa cells were treated with varying concentrations of 4-OI for 24 hours, and the subcellular location of the reporter protein was assessed. Confocal microscopy demonstrated that 4-OI treatment caused accumulation of the reporter protein in the nucleus, as shown for 250 µM (Fig. 3A). Quantification of the ratio of nuclear to cytoplasmic GFP upon treatment with a range of 4-OI revealed a dose-dependent increase of nuclear reporter protein upon 4-OI treatment (Fig. 3B). As translocation of this reporter protein depends on CRM1 activity, these results confirm that 4-OI indeed targets the activity of CRM1.

To support that 4-OI inhibits the nuclear export mediated by CRM1, the effect on the nucleus to cytoplasm translocation of the Rev protein of feline immunodeficiency virus (FIV) was used. The export of FIV Rev is mediated through its NES and is suggested to bind to CRM1. Previously reported for HIV, Rev shuttles to the cytoplasm in a CRM1-dependent manner upon actinomycin D treatment, while Rev remains mainly in the nucleus in the presence of an CRM1 inhibitor (20, 21). Here, in FIV Rev-GFP transfected Vero cells, Rev-GFP was predominantly found in the nucleus. Upon actinomycin D treatment of Vero cells transfected with Rev-GFP, a proportion of the Rev-GFP protein shuttles to the cytoplasm in the presence of DMSO (Fig. 3C). This was not the case after co-treatment with 100 µM 4-OI or 100 nM KPT-330, upon which Rev-GFP remained in the nucleus (Fig. 3C). These results confirm that 4-OI has a direct effect on CRM1-mediated transport of viral proteins from the nucleus to the cytoplasm.

### 4-OI inhibits CRM1-mediated nuclear export by directly modifying C528 of CRM1

**Fig 4:**
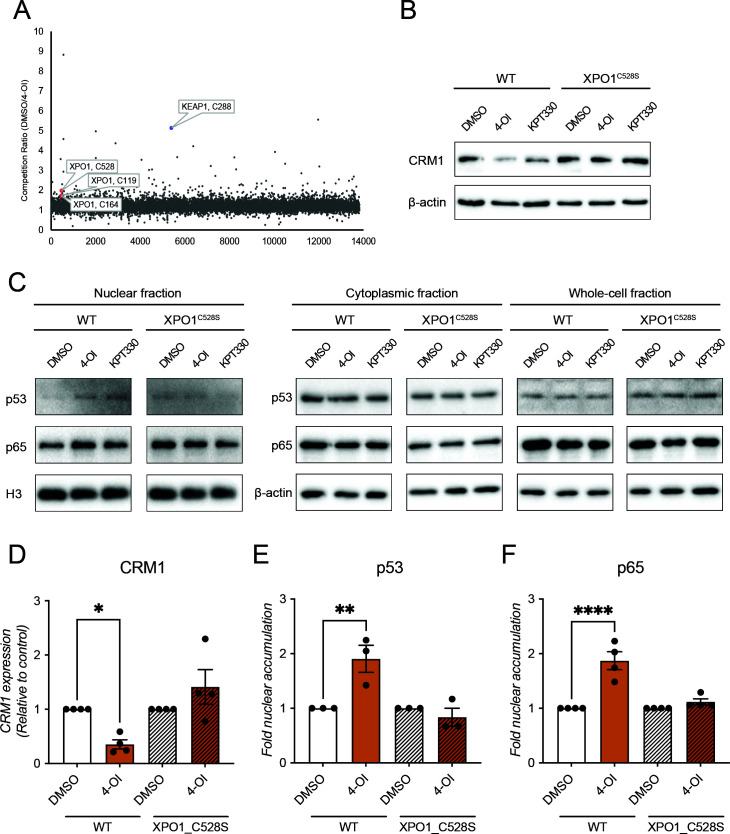
Deactivation of CRM1 by 4-OI-mediated C528 alkylation. (**A**) Mass spectrometry screening of 2,3-dicarboxypropylated cysteine residues in THP1 cells treated with 4-OI. Cells were treated with 250 µM 4-OI, and IA-DTB-based competitive cysteine profiling was performed to identify 2,3-dicarboxypropylated cysteine residues on target proteins. Highlighted are C288 on Keap1, as well as C528, C119, and C164 on CRM1. Mass spectrometry data are representative of three independent experiments. (**B**) CRM1 protein expression levels in Jurkat and Jurkat XPO1^C528S^ cells. The cells were treated with either DMSO, 250 µM 4-OI, or 25 nM KPT-330 for 24 hours. CRM1 protein expression levels were assessed by Western blotting, and β-actin was used as loading control. Blots are representative of four individual experiments. (**C**) Expression levels of p65 and p53 protein in nucleus, cytoplasm, and whole-cell fractions of Jurkat and Jurkat XPO1^C528S^ cells upon treatment. The cells were treated with DMSO, 250 µM 4-OI, or 100 nM KPT-330 for 2 hours, and nuclear-cytoplasmic fractionation was performed. H3 was used as loading control for nuclear fractions and β-actin for cytoplasmic and whole-cell fractions. Blots are representative of four (p65) or three individual experiments (p53). (**D**) Densitometry of blots shown in panel B. Protein levels have been normalized relative to the untreated control of the respective cell line. (**E and F**) Densitometry of blots shown in nuclear fraction of panel C. Protein levels have been normalized relative to the untreated control of the respective cell line.

Itaconate and its derivatives are known to be potent cysteine modifiers through a process called 2,3-dicarboxypropylation (9, 22), an alkylation reaction. CRM1 has recently been identified as a potential target for 2,3-dicarboxypropylation by itaconate (22), and CRM1 inhibitors such as LMB or the *N*-azolylacrylate derivative KPT-330 specifically act through alkylation of a key cysteine 528 inside the hydrophobic cargo binding cleft of CRM1 (18, 23, 24). To investigate whether CRM1 is indeed modified in this way by 4-OI, liquid chromatography with tandem mass spectrometry was performed on 4-OI-treated THP1 cells, and 2,3-dicarboxypropylation of cysteine residues throughout the proteome was assessed by iodoacetamide-desthiobiotin (IA-DTB)-based competitive cysteine profiling. The well-described 2,3-dicarboxypropylation target KEAP1 (9) was identified in this screen, while CRM1 was also detected as a target in the top 1% of hits (Fig. 4A). CRM1 was found to be modified in three positions: C119, C164, and C528, with C528 as the most significant hit of the three. C528 is located in the cargo binding pocket of CRM1, and alkylation of this residue has been shown to inhibit nuclear export of cargo (24, 25), rendering this cysteine a popular target for CRM1 inhibitors such as the ones mentioned before. To demonstrate that the inhibitory effect of 4-OI on CRM1 activity is through the observed 2,3-dicarboxypropylation of C528, a mutant Jurkat cell line specifically designed to study the specificity of CRM1 inhibitors was employed. These mutant Jurkat XPO1^C528S^ cells contain a serine instead of cysteine at position 528 (C528S) of the CRM1 protein. This substitution leaves the protein functionally intact but renders it resistant to modification by CRM1 inhibitors at this position (26). Hence, a decrease in CRM1 expression levels or reduction of CRM1-facilitated nuclear export upon 4-OI treatment should not occur in these mutant cells. Upon treatment with 4-OI, as well as with KPT-330, of both wild-type Jurkat cells and Jurkat XPO1^C528S^ cells, CRM1 expression levels were only significantly reduced in the wild-type cells (Fig. 4B and D). These results confirm that the C528S substitution in CRM1 indeed resulted in protection from both KPT-330- and 4-OI-mediated CRM1 degradation. In addition, the CRM1 cargo proteins p53 and p65 were accumulated in the nucleus of 4-OI-treated compared to DMSO-treated wild-type Jurkat cells, but not the mutant XPO1^C528S^ Jurkat cells, further confirming that 4-OI targets CRM1 through modification of this cysteine. Cytoplasmic and whole-cell fraction expression levels of p53 and p65 were unchanged (Fig. 4C, E, and F). To obtain additional proof for the effect of the C528S substitution in CRM1, expression levels of another CRM1 co-factor, cargo RAN binding protein 1 (RanBP1), was evaluated in wild-type and XPO1^C528S^ cells. Upon 4-OI treatment, protein expression levels of RanBP1 were significantly decreased in wild-type cells, but not in XPO1^C528S^ cells (Fig. S3A and B). As observed for p53 and p65, 4-OI treatment also led to RanBP1 accumulation in the nucleus in wild-type cells, but not in XPO1^C528S^ cells (Fig. S3C and D).

Altogether, these data indicate that the antiviral effect of 4-OI against IAV is based on targeting the C528 on CRM1, affecting the nuclear export of the NP of IAV, as well as other CRM1-dependent cargos, such as FIV Rev, PKI, p53 and p65.

## DISCUSSION

In recent years, the potential of itaconic acid and its derivatives to exert antiviral activities has been extensively investigated. 4-OI is a cell membrane-permeable derivative of itaconate and has been suggested to possess antiviral properties, including against influenza virus. Here, we report on the antiviral activity of 4-OI against influenza virus with a focus on the possible mechanism of action. In a previous reported study, treatment with 25 mM itaconate did not decrease IAV replication in human primary lung tissue (4). Nevertheless, the same study suggested itaconate to slightly decrease IAV replication at 20 mM in A549 cells (4). Here, 10 mM itaconate did not reduce the viral load in IAV infected A549 nor MDCK cells, in contrast to 4-OI at 100 µM. We show an antiviral effect of 4-OI against IAV in both MDCK and A549 cells, although the effect was more profound in MDCK cells. In A549 cells, higher doses of 4-OI than 100 µM were needed to achieve a profound antiviral effect against IAV. This might be due to higher expression levels of CRM1 on A549 cells (27), which could explain the requirement of higher doses of 4-OI to inactivate CRM1 activity in cancerous A549 cells. In both cell types, the effect was visible as early as 8 hours after infection, as well as after 16 hours and later after infection.

Olagnier et al. showed a broad antiviral effect of 4-OI treatment against several viruses, including SARS-CoV-2, in *in vitro* assays. Both itaconate and 4-OI are known to activate the transcription factor Nrf2 (9). The Nrf2 response was found to be suppressed in SARS-CoV-2-infected patients, suggesting that SARS-CoV-2 targets this pathway (3). Hence, 4-OI was proposed to mediate an antiviral response acting as an agonist for this antioxidant pathway; however, a distinct mechanism could not be identified. While these studies suggested that the antiviral mechanism of 4-OI involves activation of Nrf2, other mechanisms of action cannot be excluded. It is unlikely that activation of Nrf2 is the sole mechanism for the observed antiviral effect of itaconic acid, as the potent Nrf2 activator dimethyl itaconate (DI) (28) failed to restrict viral replication of IAV in a similar magnitude as 4-OI (4). In fact, a recent study suggested the antiviral properties of 4-OI to be Nrf2 independent but to interfere with IAV RNPs instead via interaction with CRM1 (29). Indeed, other antiviral mechanisms have been proposed for the activity of itaconate derivatives against influenza viruses such as targeting of the nuclear export of IAV vRNPs (13). In our study, not only the 4-OI-induced antiviral activity against IAV was confirmed, but the data also indicated that the antiviral activity of 4-OI is related to the inhibition of the CRM1-mediated nuclear export of NP. In addition, this study demonstrated that 4-OI targets the C528 residue of CRM1 via an alkylation mechanism previously described for leptomycin B^24^. Comparison between treatment with 4-OI and SINE compounds of IAV infected cells demonstrated a characteristic phenotype of CRM1 modification, such as entrapment of NES containing proteins in the nucleus upon treatment (30).

A useful cell line to study CRM1 inhibitors, the Jurkat XPO1^C528S^ cell line, was applied to assess the inhibitory effect of 4-OI on CRM1. While ideal for studying the cysteine modification induced by 4-OI, these cells were not well suited to study the effect on IAV replication as IAV cannot replicate in these cells. Therefore, the 4-OI-mediated alkylation of CRM1 was assessed using cellular cargos of CRM1, demonstrating modification of C528 to be crucial for the 4-OI-mediated inactivation of CRM1. CRM1 is modified by 4-OI in two additional positions that were not directly assessed in this study. The lack of an effect by 4-OI observed in the knock-in cells point toward C528 being the relevant cysteine, but a possible role for the other cysteines cannot be ruled out.

The involvement of Nrf2 activation as an antiviral contributor cannot also be fully excluded as this was not assessed. In addition, we were unable to investigate the potential antiviral role of endogenous itaconate in this context, as the itaconate production is restricted to cells of the myeloid lineage.

Since CRM1 function is involved in the replication cycle of many different viruses, the mechanism proposed here might explain antiviral effects of 4-OI described for other viruses besides IAV. In fact, all viruses sensitive to 4-OI treatment as reported by Olagnier et al. have been described to depend on CRM1 in some capacity for their replication (3, 31 – 34). This indicates that 4-OI could have an effect against viruses that depend on CRM1-mediated cargo export from the nucleus (3). Another example of well-characterized CRM1-mediated nuclear export has been described for FIV and HIV (35). As proof of principle, translocation of the Rev protein of FIV from the nucleus to the cytoplasm upon treatment with actinomycin D was impaired in the presence of 4-OI in a similar manner as described in previous studies for HIV Rev protein (21).

Although the antiviral effect of 4-OI against IAV was shown in A549 cells and MDCKs, the mechanism of action was also shown in HeLa, Vero, THP-1, and Jurkat cells. Based on the fact that the mechanism of action has been demonstrated in these different cell lines and on the fact that A549 cells do express very high levels of CRM1 (27), it seems likely that the mechanism of action of 4-OI is similar in A549 cells as described for the other cell lines.

These results indicate that direct modification of CRM1 by 4-OI-mediated alkylation contributes to the mechanism for antiviral activity observed against IAV as well as other viruses and confirms the previously reported antiviral properties of 4-OI. Although the efficacy as antiviral drug as well as possible side effects induced by 4-OI during antiviral treatment were not addressed in this study, the knowledge on the mechanism of action of 4-OI expands our knowledge of the mechanism of action which will be useful in the development of derivatives of 4-OI that could have clinical potential in treatment of viral infections where inflammation will also be a useful process to target.

## MATERIALS AND METHODS

### Cell culture

Human lung adenocarcinoma A549 cells were cultured in Ham-F12 (Gibco) supplemented with 10% fetal bovine serum (FBS, Sigma-Aldrich) and 100 U/mL penicillin-streptomycin-glutamine (Pen-Strep-Glut, Lonza) mixture. MDCK cells were cultured in Eagle’s minimal essential medium (EMEM; Lonza) plus 10% FBS, 1% Hepes 1 M, 1% sodium bicarbonate (Lonza), 1× non-essential amino acid solution, and 100 U/mL Pen-Strep-Glut mixture. A subclone 118 of Vero-WHO cells (Vero-118 (36) was cultured in Iscove’s modified Dulbecco’s medium (Lonza) supplemented with 10% FBS, and 100 U/mL Pen-Strep-Glut mixture. Jurkat cells were cultured in Roswell Park Memorial Institute (RPMI) 1640 Medium (Gibco), supplemented with 10% FBS, 100 U/mL Pen-Strep, and 1% Hepes 1 M (all Gibco). All cells were cultured at 37°C and 5% CO_2_. Monocytic THP-1 cells were cultured in RPMI 1640 (Gibco), supplemented with 10% FBS, 0.05 mM 2-mercaptoethanol (Gibco), and 100 U/mL Pen-Strep (Sigma-Aldrich).

### Viruses

Pandemic influenza A/H1N1 virus (isolate A/Netherlands/602/2009) and seasonal influenza A/NL/213/03 H3N2 virus (isolate A/Netherlands/213/2003) were generated as previously reported (37, 38). Recombinant influenza A/WSN/33 (H1N1) was generated as previously described (39).

### Virus titrations

Samples were titrated in quadruplicates in MDCK cells and seeded confluently in 96-well plates. Cells were inoculated with 10-fold serial dilutions of the sample in serum-free cell culture EMEM with IAV A/H1N1 and A/H3N2 virus or IAV A/WSN/33 virus, in the presence or absence of 0.7 µg/mL TPCK trypsin (Merck, T1426), respectively. Three days post inoculation, supernatants of infected cell cultures were tested for agglutination using turkey erythrocytes, with observed hemagglutination in the presence of the virus in the supernatant. Endpoint infectious viral titer (TCID_50_/mL) was calculated from quadruplicates for each individual sample using the Spearman-Karber method.

### Plasmids, reagents and antibodies

Plasmid expressing Rev-GFP was used in a similar manner as described before (40). See Table 1 for antibodies used in Western blotting and confocal microscopy experiments and Table 2 for other reagents.

**TABLE 1:** Antibodies used in Western blotting and confocal microscopy experiments

Target	Clone	Source	Article number
CRM1	D6V7N	Cell Signaling Technology	46249S
CRM1	5G3	Thermo Fisher	MA5-27879
GAPDH	GA1R	Thermo Fisher	MA5-15738
GAPDH	Polyclonal	Life Technologies	PA1987
H3	D1H2	Cell Signaling Technology	4499S
Influenza A NP-FITC labeled	D67J	Life Technologies	MA17322
Lamin B1	Polyclonal	Life Technologies	PA519468
Mouse IgG (H + L)	Polyclonal	Jackson ImmunoResearch	115-035-146
Mouse IgG (H + L)-AF488 labeled	Polyclonal	Thermo Fisher	A-11001
Mouse IgG2b-Alexa 594 labeled	Polyclonal	Thermo Fisher	A21145
Mouse immunoglobulins-HRP labeled	Polyclonal	Agilent Technologies	P026002-2
IAV NP	HB65	ATCC, Wesel, Germany	–
p53	Polyclonal	Cell Signaling Technology	9282S
p65	D14E12	Cell Signaling Technology	8242S
RanBP1	Polyclonal	Thermo Fisher	PA1080
Actin-CF 633 conjugate	Polyclonal	Santa Cruz Biotechnology	SC-363796
Rabbit IgG (H + L)	Polyclonal	Jackson ImmunoResearch	111-035-144
Rabbit immunoglobulins-HRP labeled	Polyclonal	Agilent technologies	P0217
β-Actin	BA3R	Thermo Fisher	MA5-15739
β-Actin-HRP labeled	C4	Santa Cruz Biotechnology	SC-47778 HRP

**TABLE 2:** Reagents used for cell treatment

Clone	Source	Article number
4-OI	R&D Systems	6662
Actinomycin D	Merck	A1410
Itaconate	Merck	I29204
KPT-330	Selleck Chemicals	S7252

### CRM1 activity assay

HeLa cells stably expressing the XPO1-dependent NLSSV40-AcGFP-NESPKI reporter were cultured as described previously (41). To assess the CRM1-mediated nuclear export function, reporter cells were seeded at 8,000 cells per well in 96-well tissue culture plates (Switzerland). The next day, cells were treated with a serial dilution of 4-OI or DMSO for 24 hours, fixated, and counterstained with DAPI. Fluorescence was imaged on an ArrayScan XTI High Content Reader (Thermo Fisher Scientific). Nuclear and cytoplasmic compartments were segmented, and their average pixel intensities in the green channel were quantitated employing the HCS Studio software (Thermo Fisher Scientific). GraphPad Prism was used for dose-response curve fitting based on the percentage of cells having a predominant nuclear localization of the reporter construct. A ratio of nuclear to cytoplasmic signal equal or above 1.4 was considered predominantly nuclear. Representative images were taken by confocal microscopy on a Leica TCS SP5 confocal microscope (Leica Microsystems), employing an HCX PL APO 63× (NA 1.2) water immersion objective.

### Flow cytometry viability assay

MDCK and A549 cells were treated for 48 hours with 4-OI or DMSO. Cells and supernatant were collected by centrifugation at 300 × *g* for 5 min. Cells were stained for 15 min at 4°C with LIVE/DEAD Fixable Red Dead Cell Stain Kit. Cells were washed twice with phosphate-buffered saline (PBS), 0.5% bovine serum albumin (BSA), and 2 mM EDTA. Readout was assessed by flow cytometry with BD FACS Lyric. Dose dependency toxicity of 4-OI was normalized to the vehicle DMSO.

### Quantitative profiling of 4-OI modified cysteinome in THP1 cells

The cysteinome in 4-OI-treated THP-1 was assessed as previously described (42). In short, THP-1 cells were seeded into 6-cm Petri dish (4 million per dish) in 5-mL RPMI 1640 culture medium and treated with DMSO, 125 µM 4-OI, or 250 µM 4-OI, respectively, for 16 hours at 37°C and 5% CO_2_. Ten dishes of cells were treated (four dishes with DMSO, three with 125 µM 4-OI, and three with 250 µM 4-OI) in each replicate experiment for utilizing TMT-10plex isobaric label reagent (Thermo Fisher Scientific) (42). Carbamidomethylation of cysteine residues was set as fixed modification, while oxidation of methionine residues, acetylation of protein N-term, and IA-DTB on cysteine residues were set as variable modifications. Competition ratio was calculated by dividing the TMT reporter ion intensities from control channel (DMSO) by the 4-OI-treated channel.

### Cellular fractionation

To separate nuclear and cytoplasmic fractions of harvested cells, NP-40 cell lysis buffer (Invitrogen) was used according to manufacturer’s instructions. Additionally, the protocol previously described by Suzuki et al. was used to achieve whole-cell, cytoplasmic, and nuclear fractions (43).

### Western blot assay

Cells were washed with PBS at 4°C and lysed in ice-cold RIPA Lysis Buffer (Thermo Fisher) containing cOmplete Mini EDTA-free Protease and PhosSTOP phosphatase Inhibitors (Merck). Total cell lysates were collected and heated at 95°C for 5 min. Samples were resolved on Mini-PROTEAN 10 or 12% sodium dodecyl sulphate-polyacrylamide gel electrophoresis gels (Bio-Rad). Proteins were transferred onto a methanol-activated 0.2-µm polyvinylidene difluoride membrane via a wet transfer system. Subsequently, membranes were blocked with 5%, wt/vol, milk (Campina) and 0.1% Tween-20 in PBS for 1 hour at room temperature (RT), washed, and incubated with primary antibody in 5%, wt/vol, milk in PBS with 0.1% Tween-20 overnight at 4°C. All primary antibody incubations were followed by incubation with secondary horseradish peroxidase (HRP)-conjugated antibodies in 5% milk and 0.1% Tween-20 in PBS for 1 hour at RT on a ChemiDoc MP Imaging System (Bio-Rad). Primary antibodies were used as follows: anti-CRM1 (MA5-27879, 1:1,000), anti-NP (HB65, 1:500), anti-Lamin B1 (PA519468, 1:1000), and anti-GAPDH (PA1987, 1:1,000), in MDCKs (Fig. 2B). Secondary antibodies were anti-rabbit HRP (P0217, 1:1,000) and anti-mouse HRP (P026002-2, 1:1,000). In Jurkat cells (Fig. 4B and C), primary antibodies anti-p53 (9282S), anti-p65 (8242S), and anti-CRM1(46249S), anti-β-actin (MA5-15739), and anti-H3 (4499S) were used. Secondary antibodies were HRP-conjugated goat-anti-rabbit (111-035-144) and goat-anti-mouse (115-035-146). Antibodies are listed in Table 1.

### Immunofluorescence microscopy

Cells were seeded at 2–4 × 10^5^ cells per well on coverslips coated with 0.1% gelatin in PBS (G1393, Merck) in 24-well plates. The following day, cells were inoculated at the indicated MOIs for 1 hour, then washed thrice with PBS, and subsequently cultured in EMEM with 100 U/mL Pen-Strep-Glut mixture for the MDCK cells until designated timepoint. Then, coverslips were washed twice with PBS and fixed with 4% paraformaldehyde at RT. Subsequently, samples were washed twice with PBS, and coverslips were stored at 4°C until staining. Permeabilization was performed with 100-µL PBS and 0.1% Triton X-100 per well for 10 min at RT. Cells were washed once with PBS and blocked with PBS 10% normal goat serum (NGS) for 1 hour. For single staining (Fig. 2A), coverslips were incubated with 50-µL primary antibody HB65 at 1:400, followed by an incubation with secondary antibody anti-mouse IgG (A-11001) at 1:500. In co-stainings (Fig. 2C), primary antibodies were anti-NP, MA17322 at 1:250, and anti-CRM1, MA5-27879 at 1:500, respectively, in PBS 10% NGS for 2 hours. After three PBS washes, coverslips were incubated in 50 µL of secondary antibody anti-mouse IgG2b, A21145 at 1:500, in PBS 10% NGS for 1 hour. After consequent PBS washes, nuclei were stained with Hoechst 20 µM (33342, Life Technologies) in PBS. Finally, coverslips were mounted using ProLong Gold Antifade Mountant solution (Invitrogen). Images were taken on an LSM700 confocal microscope, using ZEN software (Zeiss). Remaining analysis was performed using Image J software.

## AFTER EPUB

*[This article was published on 12 October 2023 with an incorrect e-mail address listed for Hauke J. Weiss in the corresponding-author footnote. The footnote was updated in the current version, posted on 31 October 2023.]*

## SUPPLEMENTAL MATERIAL

Fig. S1 jvi.01325-23-s0001.pdf Treatment optimization for 4-OI.

Supplemental Figure legends jvi.01325-23-s0002.docx Legends of Fig. S1 to S3.

Fig. S2 jvi.01325-23-s0003.pdf Effect of 4-OI on NP and CRM1 localization.

Fig. S3 jvi.01325-23-s0004.pdf Effect of 4-OI mediated CRM1 inactivation on RanBP1.

ASM does not own the copyrights to Supplemental Material that may be linked to, or accessed through, an article. The authors have granted ASM a non-exclusive, world-wide license to publish the Supplemental Material files. Please contact the corresponding author directly for reuse.

## References

[1] Krammer F , Smith GJD , Fouchier RAM , Peiris M , Kedzierska K , Doherty PC , Palese P , Shaw ML , Treanor J , Webster RG , García-Sastre A . 2018. Influenza. Nat Rev Dis Primers 4:3. doi:10.1038/s41572-018-0002-y 29955068 PMC7097467

[2] Minton K . 2019. Neuronal itaconate restricts viral infection. Nat Rev Immunol 19:67. doi:10.1038/s41577-019-0121-z 30644450

[3] Olagnier D , Farahani E , Thyrsted J , Blay-Cadanet J , Herengt A , Idorn M , Hait A , Hernaez B , Knudsen A , Iversen MB , Schilling M , Jørgensen SE , Thomsen M , Reinert LS , Lappe M , Hoang H-D , Gilchrist VH , Hansen AL , Ottosen R , Nielsen CG , Møller C , van der Horst D , Peri S , Balachandran S , Huang J , Jakobsen M , Svenningsen EB , Poulsen TB , Bartsch L , Thielke AL , Luo Y , Alain T , Rehwinkel J , Alcamí A , Hiscott J , Mogensen TH , Paludan SR , Holm CK . 2020. SARS-CoV2-mediated suppression of NRF2-signaling reveals potent antiviral and anti-inflammatory activity of 4-Octyl-itaconate and dimethyl fumarate. Nat Commun 11:5419. doi:10.1038/s41467-020-19363-y 33087717 PMC7578803

[4] Sohail A , Iqbal AA , Sahini N , Chen F , Tantawy M , Waqas SFH , Winterhoff M , Ebensen T , Schultz K , Geffers R , Schughart K , Preusse M , Shehata M , Bähre H , Pils MC , Guzman CA , Mostafa A , Pleschka S , Falk C , Michelucci A , Pessler F . 2022. Itaconate and derivatives reduce interferon responses and inflammation in influenza a virus infection. PLoS Pathog. 18:e1011002. doi:10.1371/journal.ppat.1011002 35025971 PMC8846506

[5] Michelucci A , Cordes T , Ghelfi J , Pailot A , Reiling N , Goldmann O , Binz T , Wegner A , Tallam A , Rausell A , Buttini M , Linster CL , Medina E , Balling R , Hiller K . 2013. Immune-responsive gene 1 protein links metabolism to immunity by catalyzing itaconic acid production. Proc Natl Acad Sci U S A 110:7820–7825. doi:10.1073/pnas.1218599110 23610393 PMC3651434

[6] Liao S-T , Han C , Xu D-Q , Fu X-W , Wang J-S , Kong L-Y . 2019. 4-Octyl itaconate inhibits aerobic glycolysis by targeting GAPDH to exert anti-inflammatory effects. Nat Commun 10:5091. doi:10.1038/s41467-019-13078-5 31704924 PMC6841710

[7] Hooftman A , Angiari S , Hester S , Corcoran SE , Runtsch MC , Ling C , Ruzek MC , Slivka PF , McGettrick AF , Banahan K , Hughes MM , Irvine AD , Fischer R , O’Neill LAJ . 2020. The immunomodulatory metabolite itaconate modifies NLRP3 and inhibits inflammasome activation. Cell Metab 32:468–478. doi:10.1016/j.cmet.2020.07.016 32791101 PMC7422798

[8] Bambouskova M , Gorvel L , Lampropoulou V , Sergushichev A , Loginicheva E , Johnson K , Korenfeld D , Mathyer ME , Kim H , Huang L-H , Duncan D , Bregman H , Keskin A , Santeford A , Apte RS , Sehgal R , Johnson B , Amarasinghe GK , Soares MP , Satoh T , Akira S , Hai T , de Guzman Strong C , Auclair K , Roddy TP , Biller SA , Jovanovic M , Klechevsky E , Stewart KM , Randolph GJ , Artyomov MN . 2018. Electrophilic properties of itaconate and derivatives regulate the IκBζ–ATF3 inflammatory axis. Nature 556:501–504. doi:10.1038/s41586-018-0052-z 29670287 PMC6037913

[9] Mills EL , Ryan DG , Prag HA , Dikovskaya D , Menon D , Zaslona Z , Jedrychowski MP , Costa ASH , Higgins M , Hams E , Szpyt J , Runtsch MC , King MS , McGouran JF , Fischer R , Kessler BM , McGettrick AF , Hughes MM , Carroll RG , Booty LM , Knatko EV , Meakin PJ , Ashford MLJ , Modis LK , Brunori G , Sévin DC , Fallon PG , Caldwell ST , Kunji ERS , Chouchani ET , Frezza C , Dinkova-Kostova AT , Hartley RC , Murphy MP , O’Neill LA . 2018. Itaconate is an anti-inflammatory metabolite that activates NRF2 via alkylation of KEAP1. Nature 556:113–117. doi:10.1038/nature25986 29590092 PMC6047741

[10] Lampropoulou V , Sergushichev A , Bambouskova M , Nair S , Vincent EE , Loginicheva E , Cervantes-Barragan L , Ma X , Huang SC-C , Griss T , Weinheimer CJ , Khader S , Randolph GJ , Pearce EJ , Jones RG , Diwan A , Diamond MS , Artyomov MN . 2016. Itaconate links inhibition of succinate dehydrogenase with macrophage metabolic remodeling and regulation of inflammation. Cell Metab 24:158–166. doi:10.1016/j.cmet.2016.06.004 27374498 PMC5108454

[11] ElAzzouny M , Tom CTMB , Evans CR , Olson LL , Tanga MJ , Gallagher KA , Martin BR , Burant CF . 2017. Dimethyl itaconate is not metabolized into itaconate intracellularly. J Biol Chem 292:4766–4769. doi:10.1074/jbc.C117.775270 28188288 PMC5377792

[12] Swain A , Bambouskova M , Kim H , Andhey PS , Duncan D , Auclair K , Chubukov V , Simons DM , Roddy TP , Stewart KM , Artyomov MN . 2020. Comparative evaluation of itaconate and its derivatives reveals divergent inflammasome and type I interferon regulation in macrophages. Nat Metab 2:594–602. doi:10.1038/s42255-020-0210-0 32694786 PMC7378276

[13] Sethy B , Hsieh C-F , Lin T-J , Hu P-Y , Chen Y-L , Lin C-Y , Tseng S-N , Horng J-T , Hsieh P-W . 2019. Design, synthesis, and biological evaluation of itaconic acid derivatives as potential anti-influenza agents. J Med Chem 62:2390–2403. doi:10.1021/acs.jmedchem.8b01683 30753063

[14] Qin W , Qin K , Zhang Y , Jia W , Chen Y , Cheng B , Peng L , Chen N , Liu Y , Zhou W , Wang Y-L , Chen X , Wang C . 2019. S-glycosylation-based cysteine profiling reveals regulation of glycolysis by itaconate. Nat Chem Biol 15:983–991. doi:10.1038/s41589-019-0323-5 31332308

[15] Watanabe K , Takizawa N , Katoh M , Hoshida K , Kobayashi N , Nagata K . 2001. Inhibition of nuclear export of ribonucleoprotein complexes of influenza virus by leptomycin B. Virus Res 77:31–42. doi:10.1016/s0168-1702(01)00263-5 11451485

[16] Uddin MH . 2020. Exportin 1 inhibition as antiviral therapy. Drug Discov Today 3:54–67. doi:10.1016/j.drudis.2020.06.014 PMC7305737 32569833

[17] Perwitasari O , Johnson S , Yan X , Howerth E , Shacham S , Landesman Y , Baloglu E , McCauley D , Tamir S , Tompkins SM , Tripp RA . 2014. Verdinexor, a novel selective inhibitor of nuclear export, reduces influenza A virus replication in vitro and in vivo. J Virol 88:10228–10243. doi:10.1128/JVI.01774-14 24965445 PMC4136318

[18] Sun Q , Carrasco YP , Hu Y , Guo X , Mirzaei H , Macmillan J , Chook YM . 2013. Nuclear export inhibition through covalent conjugation and hydrolysis of leptomycin B by CRM1. Proc Natl Acad Sci U S A 110:1303–1308. doi:10.1073/pnas.1217203110 23297231 PMC3557022

[19] Van Neck T , Pannecouque C , Vanstreels E , Stevens M , Dehaen W , Daelemans D . 2008. Inhibition of the CRM1-mediated nucleocytoplasmic transport by N-azolylacrylates: structure–activity relationship and mechanism of action. Bioorg Med Chem 16:9487–9497. doi:10.1016/j.bmc.2008.09.051 18835718

[20] Otero GC , Harris ME , Donello JE , Hope TJ . 1998. Leptomycin B inhibits equine infectious anemia virus Rev and feline immunodeficiency virus Rev function but not the function of the hepatitis B virus posttranscriptional regulatory element. J Virol 72:7593–7597. doi:10.1128/JVI.72.9.7593-7597.1998 9696859 PMC110012

[21] Lee CH , Chang SC , Wu CHH , Chang MF . 2001. A novel chromosome region maintenance 1-independent nuclear export signal of the large form of hepatitis delta antigen that is required for the viral assembly. J Biol Chem 276:8142–8148. doi:10.1074/jbc.M004477200 11076934

[22] Qin W , Zhang Y , Tang H , Liu D , Chen Y , Liu Y , Wang C . 2020. Chemoproteomic profiling of itaconation by bioorthogonal probes in inflammatory macrophages. J Am Chem Soc 142:10894–10898. doi:10.1021/jacs.9b11962 32496768

[23] Daelemans D , Afonina E , Nilsson J , Werner G , Kjems J , De Clercq E , Pavlakis GN , Vandamme A-M . 2002. A synthetic HIV-1 Rev inhibitor interfering with the CRM1-mediated nuclear export. Proc Natl Acad Sci U S A 99:14440–14445. doi:10.1073/pnas.212285299 12374846 PMC137902

[24] Kudo N , Matsumori N , Taoka H , Fujiwara D , Schreiner EP , Wolff B , Yoshida M , Horinouchi S . 1999. Leptomycin B inactivates CRM1/Exportin 1 by covalent modification at a cysteine residue in the central conserved region. Proc Natl Acad Sci U S A 96:9112–9117. doi:10.1073/pnas.96.16.9112 10430904 PMC17741

[25] Girirajan S , Campbell C , Eichler E . 2011. Leptomycin B alters the subcellular distribution of CRM1 (Exportin 1). Physiol Behav 176:139–148.

[26] Neggers JE , Vercruysse T , Jacquemyn M , Vanstreels E , Baloglu E , Shacham S , Crochiere M , Landesman Y , Daelemans D . 2015. Identifying drug-target selectivity of small-molecule CRM1/XPO1 inhibitors by CRISPR/Cas9 genome editing. Chem Biol 22:107–116. doi:10.1016/j.chembiol.2014.11.015 25579209

[27] Chutiwitoonchai N , Aida Y . 2016. NXT1, a novel influenza A NP binding protein, promotes the nuclear export of NP via a CRM1-dependent pathway. Viruses 8:1–15. doi:10.3390/v8080209 PMC4997571 27483302

[28] Zhang Y , Zhou Y-J , Tang J-S , Lan J-Q , Kang Y-Y , Wu L , Peng Y . 2022. A comparison study between dimethyl itaconate and dimethyl fumarate in electrophilicity, NRF2 activation, and anti-inflammation in vitro. J Asian Nat Prod Res 24:577–588. doi:10.1080/10286020.2021.1949303 34292106

[29] Waqas FH , Shehata M , Elgaher WAM , Lacour A , Kurmasheva N , Begnini F , Kiib AE , Dahlmann J , Chen C , Pavlou A , Poulsen TB , Merkert S , Martin U , Olmer R , Olagnier D , Hirsch AKH , Pleschka S , Pessler F . 2023. NRF2 activators inhibit influenza A virus replication by interfering with nucleo-cytoplasmic export of viral RNPs in an NRF2-independent manner. PLoS Pathog 19:e1011506. doi:10.1371/journal.ppat.1011506 37459366 PMC10374058

[30] Turner JG , Dawson J , Emmons MF , Cubitt CL , Kauffman M , Shacham S , Hazlehurst LA , Sullivan DM . 2013. CRM1 inhibition sensitizes drug resistant human myeloma cells to topoisomerase II and proteasome inhibitors both in vitro and ex vivo. J Cancer 4:614–625. doi:10.7150/jca.7080 24155773 PMC3805989

[31] Kashyap T , Murray J , Walker CJ , Chang H , Tamir S , Hou B , Shacham S , Kauffman MG , Tripp RA , Landesman Y . 2021. Selinexor, a novel selective inhibitor of nuclear export, reduces SARS-CoV-2 infection and protects the respiratory system in vivo. Antiviral Res 192:105115. doi:10.1016/j.antiviral.2021.105115 34157321 PMC8213878

[32] Williams P , Verhagen J , Elliott G . 2008. Characterization of a CRM1-dependent nuclear export signal in the C terminus of herpes simplex virus type 1 tegument protein Ul47. J Virol 82:10946–10952. doi:10.1128/JVI.01403-08 18715912 PMC2573212

[33] Slezak K , Michalik M , Kowalczyk A , Rokita H . 2004. YY1 is recruited to the cytoplasm of vaccinia virus-infected human macrophages by the CRM1 system. Virus Res 102:177–184. doi:10.1016/j.virusres.2004.01.028 15084399

[34] Zhao Z , Tao M , Han W , Fan Z , Imran M , Cao S , Ye J . 2021. Nuclear localization of Zika virus NS5 contributes to suppression of type I interferon production and response. J Gen Virol 102:001376. doi:10.1099/jgv.0.001376 31859616 PMC8515865

[35] Elinav H , Wu Y , Coskun A , Hryckiewicz K , Kemler I , Hu Y , Rogers H , Hao B , Ben Mamoun C , Poeschla E , Sutton R . 2012. Human CRM1 augments production of infectious human and feline immunodeficiency viruses from murine cells. J Virol 86:12053–12068. doi:10.1128/JVI.01970-12 22933280 PMC3486471

[36] Kuiken T , van den Hoogen BG , van Riel DAJ , Laman JD , van Amerongen G , Sprong L , Fouchier RAM , Osterhaus ADME . 2004. Experimental human metapneumovirus infection of cynomolgus macaques (Macaca fascicularis) results in virus replication in ciliated epithelial cells and pneumocytes with associated lesions throughout the respiratory tract. Am J Pathol 164:1893–1900. doi:10.1016/S0002-9440(10)63750-9 15161626 PMC1615765

[37] Schrauwen EJA , Herfst S , Chutinimitkul S , Bestebroer TM , Rimmelzwaan GF , Osterhaus ADME , Kuiken T , Fouchier RAM . 2011. Possible increased pathogenicity of pandemic (H1N1) 2009 influenza virus upon reassortment. Emerg Infect Dis 17:200–208. doi:10.3201/eid1702.101268 21291589 PMC3204778

[38] Munster VJ . 2009. Pathogenesis and transmission of swine-origin 2009 A(H1N1) influenza virus in ferrets. Science 503:481–483. doi:10.1126/science.1177127 PMC4814155 19574348

[39] de Wit E , Spronken MIJ , Bestebroer TM , Rimmelzwaan GF , Osterhaus ADME , Fouchier RAM . 2004. Efficient generation and growth of influenza virus A/PR/8/34 from eight cDNA fragments. Virus Res 103:155–161. doi:10.1016/j.virusres.2004.02.028 15163504

[40] Stauber R , Gaitanaris GA , Pavlakis GN . 1995. Analysis of trafficking of Rev and transdominant Rev proteins in living cells using green fluorescent protein fusions: transdominant Rev blocks the export of Rev from the nucleus to the cytoplasm1. Virology 213:439–449. doi:10.1006/viro.1995.0016 7491768

[41] Vercruysse T , De Bie J , Neggers JE , Jacquemyn M , Vanstreels E , Schmid-Burgk JL , Hornung V , Baloglu E , Landesman Y , Senapedis W , Shacham S , Dagklis A , Cools J , Daelemans D . 2017. The second-generation Exportin-1 inhibitor KPT-8602 demonstrates potent activity against acute lymphoblastic leukemia. Clin Cancer Res 23:2528–2541. doi:10.1158/1078-0432.CCR-16-1580 27780859

[42] Runtsch MC , Angiari S , Hooftman A , Wadhwa R , Zhang Y , Zheng Y , Spina JS , Ruzek MC , Argiriadi MA , McGettrick AF , Mendez RS , Zotta A , Peace CG , Walsh A , Chirillo R , Hams E , Fallon PG , Jayamaran R , Dua K , Brown AC , Kim RY , Horvat JC , Hansbro PM , Wang C , O’Neill LAJ . 2022. Itaconate and itaconate derivatives target JAK1 to suppress alternative activation of macrophages. Cell Metab 34:487–501. doi:10.1016/j.cmet.2022.02.002 35235776

[43] Suzuki K , Bose P , Leong-Quong RY , Fujita DJ , Riabowol K . 2010. REAP: a two minute cell fractionation method. BMC Res Notes 3:294. doi:10.1186/1756-0500-3-294 21067583 PMC2993727

