# Supplementary figures and images for: 4-Octyl itaconate reduces influenza A replication by targeting the nuclear export protein CRM1
Source: J Virol. 2023 Oct 12;97(10):e01325-23. doi: 10.1128/jvi.01325-23 (PMC10617539; doi:10.1128/jvi.01325-23)

Figure S1

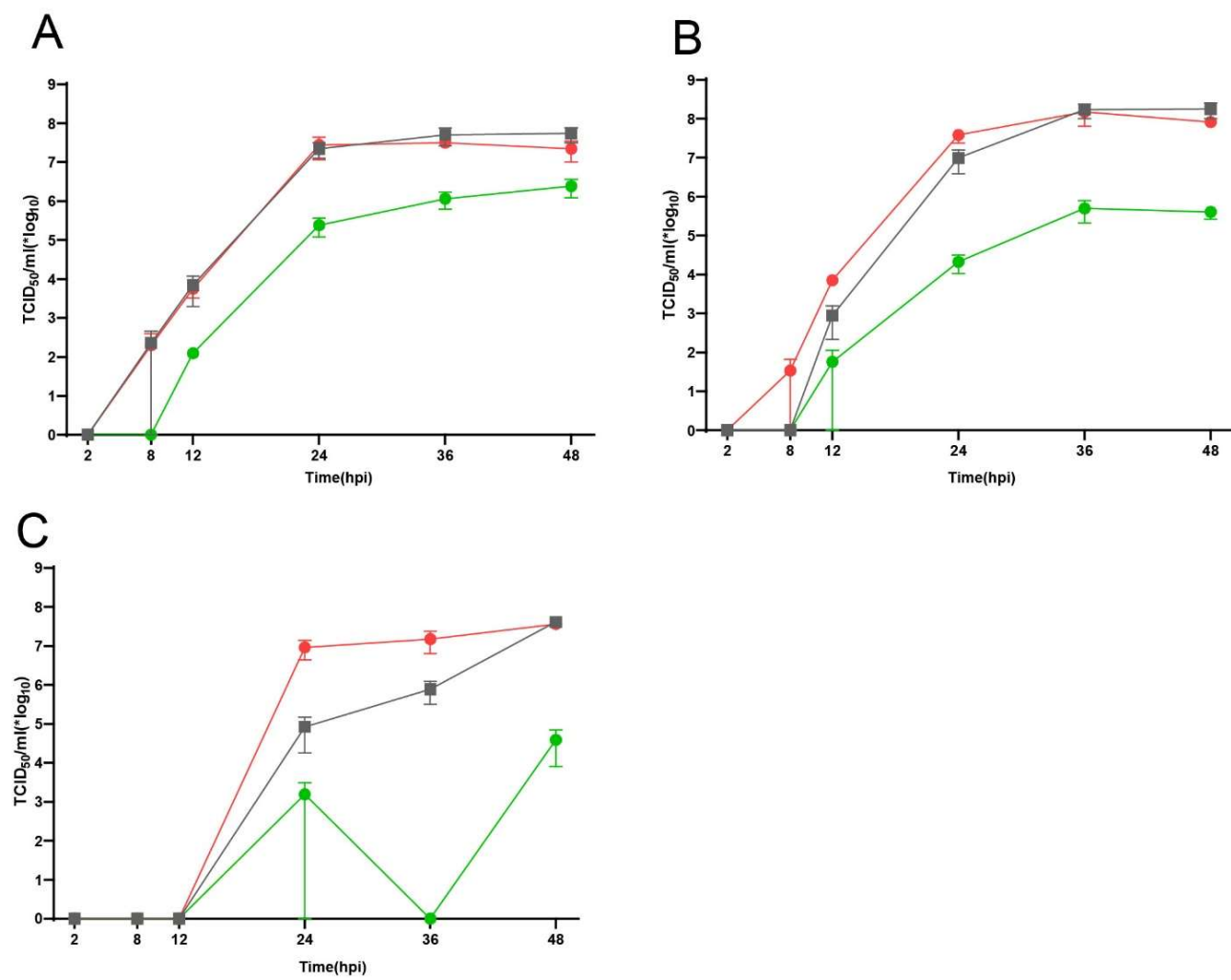

Supplement: Fig. S1 — Treatment optimization for 4-OI. [file jvi.01325-23-s0001.pdf]

**Figure S2**

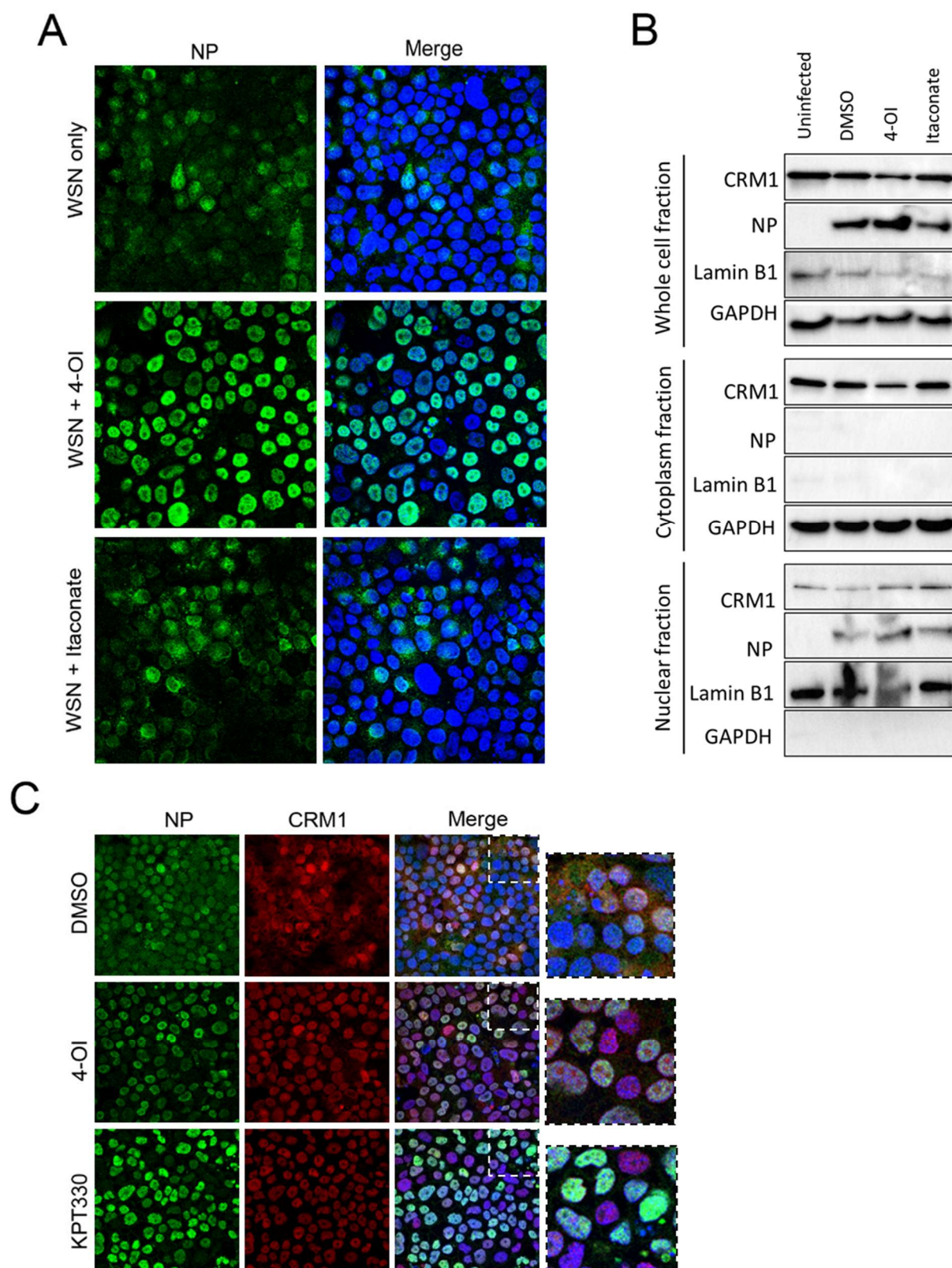

Supplement: Fig. S2 — Effect of 4-OI on NP and CRM1 localization. [file jvi.01325-23-s0003.pdf]

**Figure S3**

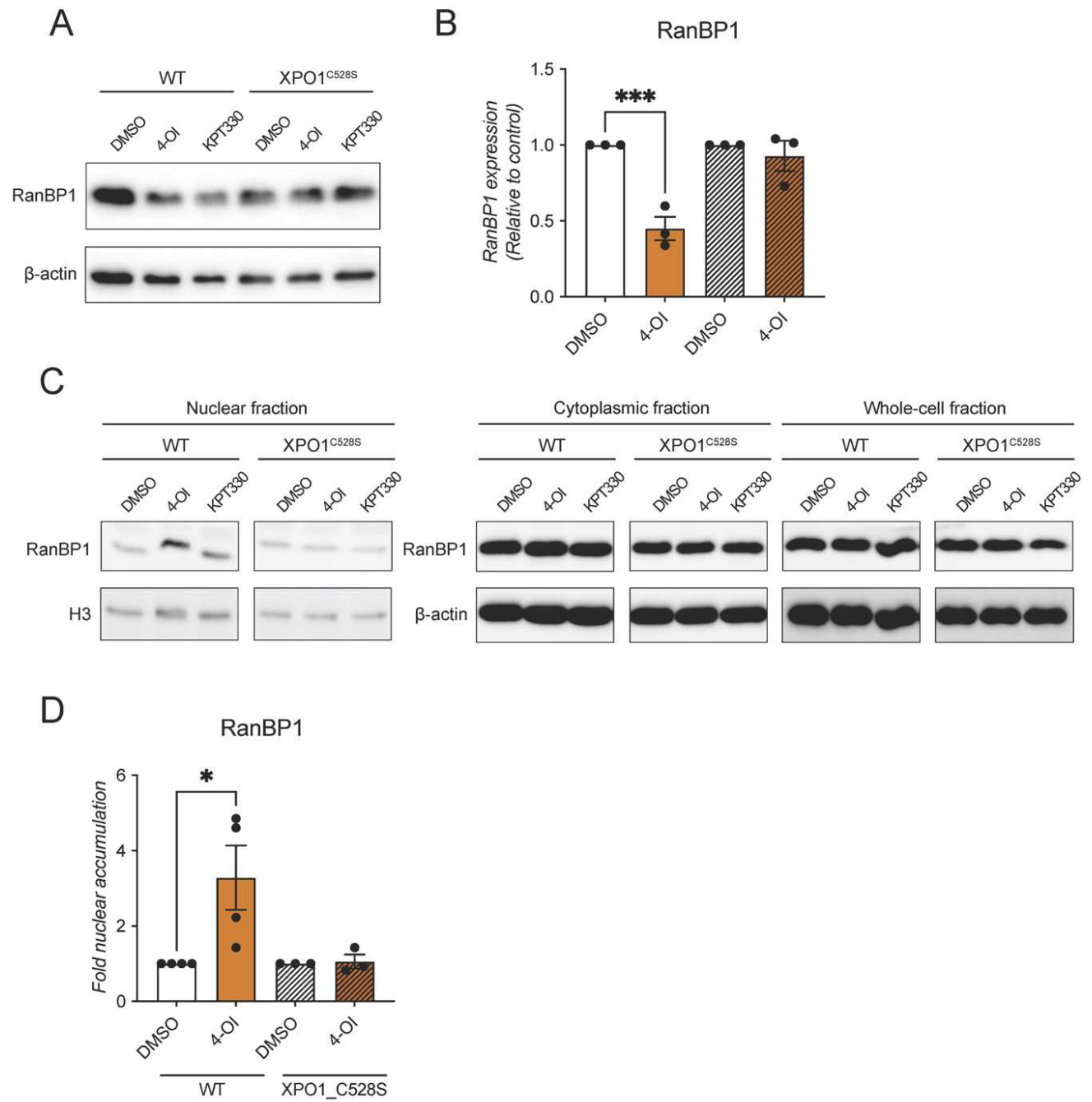

Supplement: Fig. S3 — Effect of 4-OI mediated CRM1 inactivation on RanBP1. [file jvi.01325-23-s0004.pdf]
