# Supplementary material for: 4-Octyl itaconate reduces influenza A replication by targeting the nuclear export protein CRM1
Source: J Virol. 2023 Oct 12;97(10):e01325-23. doi: 10.1128/jvi.01325-23 (PMC10617539; doi:10.1128/jvi.01325-23)
Supplement: Supplemental Figure legends — Legends of Fig. S1 to S3. [file jvi.01325-23-s0002.docx]

**Figure S1. Treatment optimization for 4-OI. A-C.** Replication kinetics on MDCK cells that were inoculated with MOI 0.01 of IAV A/NL/602/09 H1N1 (A), A/NL/213/03 H3N2 (B) and A/WSN/33 (C). Cells were only pretreated (P, grey) or pretreated and treated (P+T, green) with 100 μM 4-OI. Treated cells were compared to untreated but inoculated cells (red). Pretreatment was not sufficient to decrease the replication of the different IAV. Instead, the experimental setup of pretreatment with subsequent treatment after inoculation, was highly effective in reducing the replication of the three IAV subtypes. Replication kinetics are representative of 3 individual experiments.

**Figure S2. Effect of 4-OI on NP and CRM1 localization. A.** Confocal microscopy image of MDCK cells inoculated with IAV A/WSN/33 (MOI 1) treated with 4-OI, itaconate or left untreated. At 8 hpi, cells were fixed and stained for NP (green), to assess cellular localization. Merged depicts NP (green) together with nuclear staining Hoechst (blue). Confocal images are representative of 3 individual experiments. **B.** Protein expression levels of CRM1 and IAV NP in whole cell lysate, cytoplasmic and nuclear fractions of uninfected MDCK cells and MDCK cells inoculated with IAV A/WSN/33 (MOI 1), without treatment or treated with 100 µM 4-OI and itaconate. Nuclear marker Lamin B1 and cytoplasmic marker GAPDH were included for comparison. Images are representative of 3 individual experiments. **C.** Confocal microscopy image of MDCK cells inoculated with IAV A/WSN/33 (MOI 1) and treated with equimolar DMSO, 100 µM 4-OI, and 100 nM KPT-330. At 8hpi, cells were fixed and stained for the NP protein (green), CRM1 (red), nuclear staining Hoechst (blue) shown in Merge. Confocal images are representative of 3 individual experiments.

**Figure S3: Effect of 4-OI mediated CRM1 inactivation on RanBP1. A.** RanBP1 protein expression levels in Jurkat and Jurkat XPO1^C528S^ cells. Cells were treated with either DMSO, 250 µM 4-OI or 25 nM KPT-330 for 24 hrs. RanBP1 protein expression levels were assessed by western blotting and β-actin was used as loading control. Blots are representative of 3 individual experiments. **B.** Densitometry of blots shown in panel A. Protein levels have been normalized relative to the untreated control of the respective cell line. **C.** Expression levels of RanBP1 protein in nucleus, cytoplasm and whole cell fractions of Jurkat and Jurkat XPO1^C528S^ cells upon 4-OI treatment. The cells were treated with DMSO, 250 µM 4-OI or 100 nM KPT-330 for 2 hrs and nuclear-cytoplasmic fractionation was performed. H3 was used as loading control for nuclear fractions and β-actin for cytoplasmic and whole-cell fractions. Blots are representative of 4 (WT) or 3 (XPO1^C528S^) individual experiments. **D.** Densitometry of blots of nuclear RanBP1 are shown in panel C. Protein levels have been normalized relative to the untreated control of the respective cell line.
